# A Reference Handbook of the Diseases of Children

**Published:** 1906-04

**Authors:** 


A REFERENCE HANDBOOK OF THE DISEASES OF CHILDREN. For Students and Practitioners. By Prof. Ferdinand Fruhwald, of Vienna. Edited, with additions, by Thompson S. Westcott, M.D., Associate Professor of Diseases of Children in the University of Pennsylvania. Octavo volume of 553 pages, with 176 illustrations. Philadelphia and London: W. B. Saunders Company, 1906. Cloth, $4.50 net; half morocco, $5.50 net.

To those of the medical profession who are acquainted with Professor Fruhwald's work in the original German, it is hardly necessary to speak of the extremely valuable service the W. B. Saunders Company has performed in presenting this English translation. It must be said, however, that the translation possesses an advantage over the original—though it be the work of a leader amongst leaders in pediatric knowledge—in that the editor, Dr. Westcott, has incorporated much valuable matter, the results of his own valuable experience. With a view to making it of special service as a practical reference book, the individual diseases have been arranged alphabetically, with numerous cross-references. This is a novel feature of untold value to the busy practitioner who wishes information quickly. Special consideration has been given to symptomatology, and the prophylactic, therapeutic, and dietetic treatments have been elaborately discussed; especially is therapy treated according to the latest discoveries. The illustrations are practical and therefore excellent, nearly all being reproductions of original photographs and drawings representing cases from Professor Fruhwald's own clinic. Indeed, we can foresee for this work the same great success in this country that it has achieved in Germany.

---

From New York *Medical Journal* and Philadelphia *Medical Journal*, Consolidated. New York, July 18, 1903.

BACTERIOLOGICAL CHART, in Colors, Showing Sixty Characteristic Plates of Pathological Bacteria. New York: M. J. Breitenbach Co., 1903.

The chart embraces sixty plates, the majority of which show the characteristic bacteria, with the distinctive stainings, magnified 1,000 diameters. It includes eight different plates of *Hœmanmœba malariœ,* showing the organism at various stages of development, and in the different forms which it assumes in the various kinds of malarial fevers. In these particular plates, as well as those showing *Amœba coli,* an even greater magnification is given. The chart has evidently been brought down to a very recent date, as it embraces an admirable illustration of De Lisle and Jullien's bacillus of syphilis. The work is one of the most admirable pieces of lithographic production that we have ever seen emanate from an American house, and the fine gradation of color and variation in tints are so accurately reproduced as to make the chart of great value as well as one of an unusual degree of artistic merit. It is securely mounted in a form which enables the physician to suspend it for ready inspection.

